# Comparison of two forms of daily preventive zinc supplementation versus therapeutic zinc supplementation for diarrhea on young children’s physical growth and risk of infection: study design and rationale for a randomized controlled trial

**DOI:** 10.1186/s40795-018-0247-6

**Published:** 2018-11-29

**Authors:** K. Ryan Wessells, Kenneth H. Brown, Sengchanh Kounnavong, Maxwell A. Barffour, Guy-Marino Hinnouho, Somphou Sayasone, Charles B. Stephensen, Kethmany Ratsavong, Charles P. Larson, Charles D. Arnold, Kimberly B. Harding, Gregory A. Reinhart, Ganjana Lertmemongkolchai, Supan Fucharoen, Robin M. Bernstein, Sonja Y. Hess

**Affiliations:** 1 Program in International and Community Nutrition, Department of Nutrition, University of California, Davis, 1 Shields Avenue, Davis, CA 95616 USA; 2 Nutrition and Global Development, Bill & Melinda Gates Foundation, Seattle, WA USA; 3 National Institute of Public Health, Ministry of Health, Vientiane, Lao People’s Democratic Republic; 4 United States Department of Agriculture, Western Human Nutrition Research Center, Davis, CA USA; 5 Canadian Coalition for Global Health Research, Ottawa, Canada; 6 Nutrition International, formerly The Micronutrient Initiative, Ottawa, Canada; 7 The Mathile Institute for the Advancement of Human Nutrition, Dayton, OH USA; 8 Centre for Research and Development of Medical Diagnostics Laboratories, Faculty of Associated Medical Sciences, Khon Kaen University, Khon Kaen, Thailand; 9 Department of Anthropology, University of Colorado, Boulder, CO USA

**Keywords:** Zinc, Diarrhea, Micronutrient, Supplementation, Home-fortification, Growth, Preventive, Therapeutic

## Abstract

**Background:**

Zinc is an essential nutrient that is required for children’s normal growth and resistance to infections, including diarrhea and pneumonia, two major causes of child mortality. Daily or weekly preventive zinc supplementation has been shown to improve growth and reduce the risk of infection, while therapeutic zinc supplementation for 10–14 days is recommended for the treatment of diarrhea. The overall objective of the present study is to compare several regimens for delivering zinc to young children, both for the prevention of zinc deficiency and the treatment of diarrhea.

**Methods:**

The present study is a community-based, randomized controlled trial in the Lao People’s Democratic Republic (PDR). Three thousand, four hundred children 6–23 months of age will be randomized to one of four intervention groups (daily preventive zinc dispersible tablet, daily preventive multiple micronutrient powder, therapeutic zinc dispersible tablet for diarrhea, or placebo control); interventions will be delivered for 9 months and outcomes measured at pre-determined intervals. Primary outcomes include physical growth (length and weight), diarrhea incidence, hemoglobin and micronutrient status, and innate and adaptive immune function. Secondary outcomes include mid-upper-arm circumference, neuro-behavioral development, hair cortisol concentrations, markers of intestinal inflammation and parasite burden. Incidence of adverse events and the modifying effects of inherited hemoglobin disorders and iron status on the response to the intervention will also be examined. We will estimate unadjusted effects and effects adjusted for selected baseline covariates using ANCOVA.

**Discussion:**

Many countries are now rolling out large-scale programs to include therapeutic zinc supplementation in the treatment of childhood diarrhea, but few have established programs demonstrated to be effective in the prevention of zinc deficiency. This study will address how best to deliver supplemental zinc to prevent zinc deficiency and reduce the severity of diarrhea-related health complications.

**Trial registration:**

Trial registration identifier (NCT02428647) ; Date of registration: April 29, 2015.

## Background

Adequate zinc (Zn) nutrition is essential for optimal health and growth of young children. Multiple community-based intervention trials in low- and middle-income countries with an elevated risk of zinc deficiency have found that preventive zinc supplementation, provided in the form of a single micronutrient solution or dispersible tablet, decreases the incidence of diarrhea and acute lower respiratory infection [1–4], and likely reduces child mortality [2, 3, 5–8]. Notably, the Lancet series on maternal and child nutrition estimated that in 2011, 116,000 deaths in children under 5 years of age were attributable to zinc deficiency [9]. Apart from these effects of zinc on morbidity and mortality from common childhood infections, studies indicate that preventive zinc supplementation also increases children’s linear growth and weight gain [2, 3].

Although the beneficial impacts of daily preventive zinc supplementation have been firmly established [10], programmatic scale-up has been limited, as public health experts, governments and donors may be reluctant to support an intervention which requires daily supplementation with a single micronutrient long-term, particularly in settings where several micronutrient deficiencies often co-exist [11, 12]. Thus, multiple micronutrient interventions, especially multiple micronutrient powders (MNP), are a preferred approach to improve young children’s nutrition and health. MNP were originally designed for home-fortification of complementary foods to prevent iron deficiency and iron deficiency anemia, but often include zinc along with other micronutrients [13]. As of 2014, 50 countries were implementing large-scale programs to distribute MNP [14]. While there is consistent evidence for a beneficial impact of MNP on iron status and anemia [15], their impact on zinc status has been inconsistent [16–19] and the beneficial impact of MNP on health and growth outcomes, typically observed with preventative zinc supplementation provided in the form of a single micronutrient solution or dispersible tablet, has not been demonstrated [12]. Moreover, the evidence is inconsistent of the impact of zinc and multiple micronutrients on child development [20, 21]. Concerns about potential risks of MNP have recently been raised. MNP use has been associated with altered gut microbiota, intestinal inflammation, and an increased risk of morbidity in some studies, possibly related to the provision of supplemental iron in MNP (10–12.5 mg/d) and possibly modified by individuals’ underlying iron status [19, 22]. Thus, additional research is needed to elucidate the efficacy of MNP as a preventive zinc supplement, and to further understand the benefits and risks associated with MNP.

In addition to the aforementioned interventions designed to prevent zinc deficiency, a separate set of treatment studies has found that therapeutic zinc supplementation administered during episodes of diarrhea decreases the duration of illness among children > 6 months of age [23–26]. In response to these findings, the World Health Organization (WHO) and UNICEF now recommend administering zinc supplements (20 mg/d for 10–14 days) as adjunctive therapy with each episode of diarrhea [27]. Therapeutic zinc supplementation can be viewed as a potential opportunity to improve children’s overall zinc nutriture [1]; however, this strategy would rely on a triggering event (new episode of diarrhea) and appropriate care seeking practices and adherence, which may limit its ability to enhance population zinc status. In addition, current evidence seems to indicate that the benefit of therapeutic zinc supplementation persists only during the period of supplementation [28–30]. Additional evidence is needed to adequately characterize the comparative benefits of therapeutic zinc, given in relation to diarrhea episodes, versus preventive zinc supplements, in the form of a single micronutrient solution or dispersible tablet, or a multiple micronutrient powder.

The present study builds upon a previously published randomized controlled trial implemented in Burkina Faso, West Africa in which the effects of preventive, intermittent and therapeutic zinc supplementation regimens on the primary outcomes of diarrhea and malaria incidence and linear growth were compared among young children [31]. The findings of that study were inconclusive (and potentially inconsistent with the existing evidence base) and the authors suggested that the effects of preventive and therapeutic zinc supplementation regimens should be re-examined in populations likely to respond to zinc interventions.

This randomized controlled trial is designed to provide empirical evidence to inform global guidelines and country policies for delivering supplemental zinc to young children, both for the prevention of zinc deficiency and the treatment of diarrhea, such that a single approach could provide the maximal beneficial impact on the health and nutritional status of young children. The primary objectives of the trial are to compare the effects of daily preventive zinc supplementation (as a zinc only dispersible tablet or MNP with zinc), therapeutic zinc treatment (in relation to an episode of diarrhea) and a placebo control on: 1) physical growth (length and weight), 2) the incidence of diarrhea, 3) hemoglobin and micronutrient status, and 4) innate and adaptive immune response during the 36 week intervention period. Secondary objectives are: 1) to assess the effects of the different supplementation regimens on mid-upper arm circumference, acquisition of developmental milestones, hair cortisol concentrations, intestinal inflammation and parasite burden, and 2) to explore the possible modifying effects of inherited hemoglobin disorders (IHbD) and iron status on the effects of MNP on study outcomes. These secondary objectives will permit in-depth assessments of factors possibly contributing to benefits and risks of MNP and preventive and therapeutic zinc supplementation on growth, health and development of young children compared with a control group [32–36].

## Methods/design

### Trial administration and consenting procedures

The study protocol was first reviewed by a steering committee at the Ministry of Health (MOH) and the Ministry of Foreign Affairs in Lao People’s Democratic Republic (Lao PDR). A MOH advisory committee, a MOH coordinating committee and a technical committee were created for the purpose of reporting on study implementation and progress. The trial, including the pilot survey, was approved by the National Ethics Committee for Health Research, Ministry of Health, Lao PDR (NECHR; 040/2014, 069/2015, 039/2016), the University of California, Davis Institutional Review Board (IRB; 626187), and the Khon Kaen University, Thailand Ethics Committee in Health Research (HE572312 and HE592006). The trial is registered at https://clinicaltrials.gov/ct2/show/NCT02428647 (NCT02428647). On the day of enrollment, consent materials are presented in the Lao language in both written and oral format; materials are first presented in a group education session conducted by a medical doctor, followed by a one-on-one session with a study nurse. Written informed consent for the pilot survey and randomized controlled trial is documented with either a written signature or a fingerprint in the presence of a neutral witness, prior to enrollment in the study. Caregivers provide additional written consent for the collection and future use of biological specimens in ancillary studies (additional indicators of nutrition, growth and infections). Oral consent, documented on a verbal consent log, is obtained for participation in focus group discussions, anthropometry standardizations, community anthropometry screening, the first stool sample collection, nail sample collection and sleep assessments.

### Study design

The Lao Zinc Study is a community-based randomized, double-blind, controlled trial. Approximately 3400 infants and young children 6–23 months of age will be enrolled, and individually-randomized to one of four intervention groups, which determines the type of preventive intervention the child receives (i.e., zinc tablet, MNP, placebo tablet or placebo powder) and type of treatment for episodes of diarrhea (i.e., dispersible zinc or placebo tablets). In all groups, children will remain under observation and receive their assigned supplements for a period of 36 weeks.

### Study setting, participant eligibility criteria, and enrolment strategy

#### Setting

Khammouane Province, in Lao PDR, was selected as the study site due to a high prevalence of stunting and underweight in young children (40.8% stunting prevalence, 29.4% underweight prevalence among under-five children based on the WHO Child Growth Standards) [37], the suspected likelihood of a high prevalence of zinc deficiency [38–40], reasonably high population density and the lack of programs currently being implemented to reduce the risk of micronutrient deficiencies or treat diarrhea with therapeutic zinc supplements. Five of the ten districts within the province were selected based on the following criteria: 1) prevalence of stunting and general socio-economic conditions; 2) population density; and 3) ease of vehicular access during all seasons to the central study office in Nongbok (Khammouane Province), as well as to Nakhon Phanom, Thailand for daily transport of biochemical samples, and Vientiane, Lao PDR for regular field supervision by senior staff from the National Institute of Public Health (NIOPH).

To confirm the extent of zinc deficiency in the study area, a pilot survey was conducted among infants and young children 6–23 months in six randomly selected villages in the Nongbok and Xebangfai districts in January – February 2015. The pilot study was implemented to establish the baseline prevalence of zinc deficiency and stunting, and hence the potential for impact. The prevalence of zinc deficiency (plasma zinc concentration < 65 μg/dL) was 61.8% (*n* = 87), and did not differ between the two districts. The prevalence of stunting was 26.3% (*n* = 114), but differed significantly by district (Nongbok = 8.8%, Xebangfai = 43.9%; *P* < 0.001). Consequently, the stunting prevalence was assessed in all villages in the catchment areas of rural health centers in the Nongbok district and only villages in the catchment areas of health centers with a mean stunting prevalence > 25%, indicative of potential zinc deficiency of public health concern [41], were selected for participation. Based on the aforementioned criteria, seven of the 11 health centers were included in the trial, representing approximately 56% of the targeted children 6–23 months in the catchment areas of rural health centers in the Nongbok district. On the basis of available national and provincial data, it was expected that the three districts not included in the pilot study (Mahaxay, Xaibouathong, Yommalath) would have stunting rates similar to, or greater than that observed in Xebangfai, and were included in the randomized controlled trial without pre-screening.

#### Identification and recruitment of participants

**Fig. 1:**
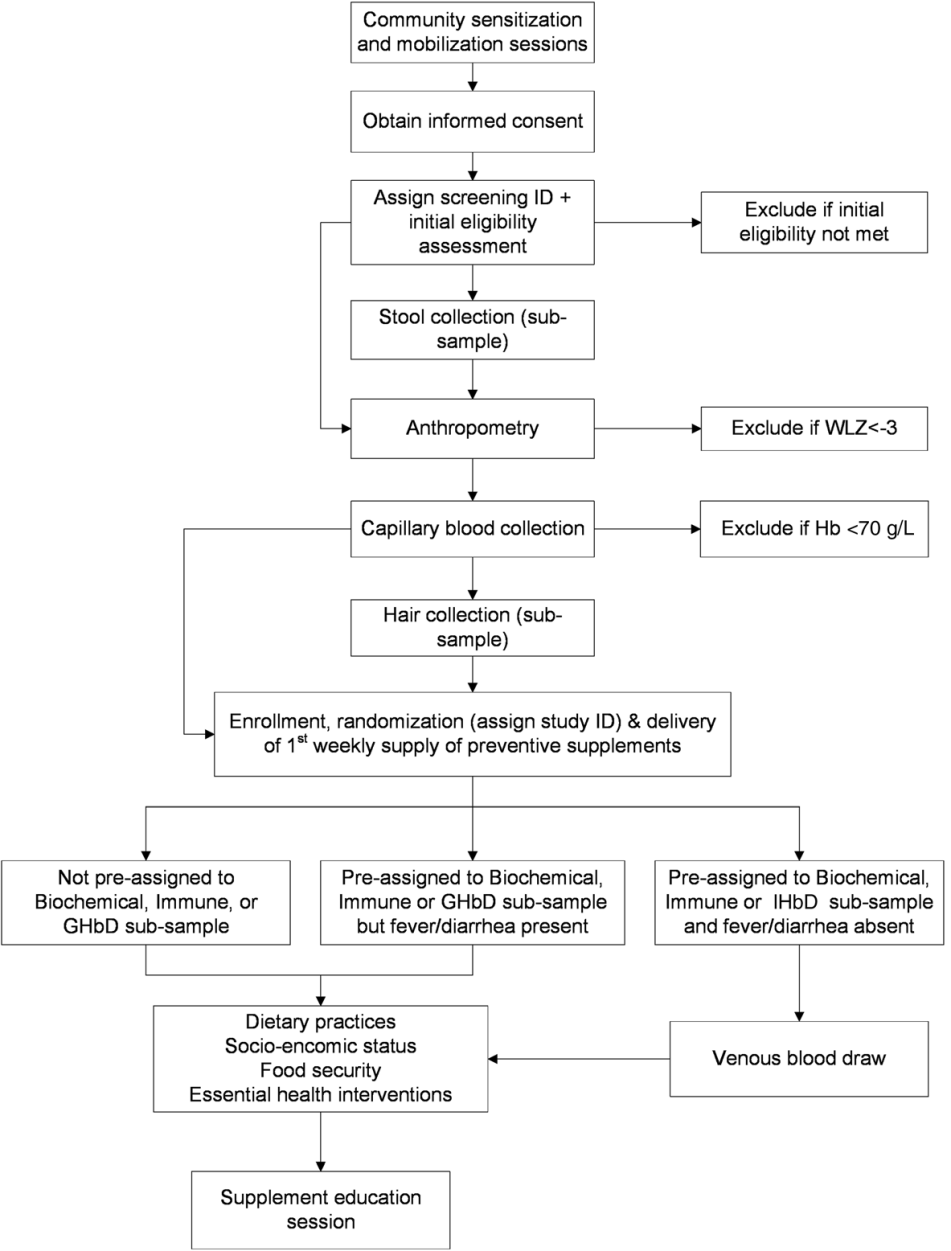
Enrollment and baseline assessments

Figure 1 depicts each step relating to the enrollment process, starting with community sensitization meetings held at the district, health center, and village levels. At the district level, meetings are held with governmental medical officers, private sector health practitioners, and pharmacists to explain the Lao Zinc Study and to solicit their cooperation. Similar meetings are held at each health center, and include all village heads and health center workers. At the village level, caregivers are invited to attend a community mobilization session at a central village location (e.g. temple) prior to the planned screening and enrollment date in their village. Mobilization and sensitization sessions are led by a medical doctor from the NIOPH and supported by representatives from the respective health centers. Topics covered at these sessions include the public health concern of micronutrient deficiencies among young children in Lao PDR, the importance of adequate dietary zinc intake for children’s health and development, an overview of the Lao Zinc Study, study eligibility criteria, the informed consent procedures (with emphasis on voluntary participation), duration of the study, study interventions and study procedures including collection of biological samples. Caregivers who express interest in enrolling their child in the trial are invited to return to a central village location on the scheduled screening date to determine if their child is eligible to participate in the trial.

#### Inclusion and exclusion criteria

Inclusion criteria are 6–23 months of age at enrollment, acceptance of weekly home visits for morbidity surveillance, planned residency within the study area for the duration of the study period (36–40 weeks), and signed informed consent from a parent or guardian. Children are ineligible for inclusion if they meet any of the following criteria: weight-for-length z-score (WLZ) < − 3 SD with respect to WHO Child Growth Standards [42], presence of bipedal edema, severe illness warranting hospital referral, congenital abnormalities potentially interfering with growth, chronic medical condition (e.g. malignancy) requiring frequent medical attention, known human immunodeficiency virus (HIV) infection of index child or child’s mother, severe anemia (hemoglobin < 70 g/L; based on testing at enrollment), currently consuming zinc supplements or current participation in any other clinical trial.

#### Randomization and blinding

The randomization scheme was generated by UC Davis statisticians using a computer-generated block randomization scheme, with randomly selected block lengths of four or eight. Each of the four study arms is identified by a unique 2-digit code and a group color, as described in more detail below. To ensure blinding of study participants, field workers, statistician and investigators of this intervention trial, each child receives a preventive product and a therapeutic supplement in case of diarrhea. Thus, caregivers, children and study personnel know which group color a child belongs to and whether the child receives a preventive tablet or a powder sachet, but not the micronutrient content of the different products. To further ensure blinding, the specific nutrient contents of the combinations of products in each of the four study arms were not explicitly detailed during fieldworker training or provided descriptions of the study. A UC Davis faculty member, unaffiliated with the study, was responsible for assigning each of the four study codes to an intervention product and communicating this information directly to the product manufacturers.

The identity of the treatment codes is stored in sealed envelopes held by the co-principal investigators (PIs) and the statistician. The envelopes will be opened only after statistical analyses of primary outcomes are completed and consensus on the interpretation of results is reached, unless required by one of the IRBs or the data safety and monitoring board (DSMB).

#### Allocation to treatment group

Eligible children in individual households are randomly assigned to one of the four intervention groups, each associated with a unique combination of preventive and therapeutic supplementation regimens. Unique randomization codes (study IDs) are sequentially assigned by a dedicated staff member, after a child is deemed eligible to participate in the study. If more than one non-twin child is eligible per household, only the younger child is enrolled in the study. The rationale for including only one child per household is to avoid intra-household correlation in disease incidence and confusion associated with different children in the same household receiving different intervention products, due to randomization at the level of the individual. In the case of twins, both children receive the same study product to avoid the risk of sharing supplements and data are collected on both children. At the end of the study, one child of the twin pair will be randomly selected for inclusion in the analyses.

### Interventions

Children are individually-randomized to one of four intervention groups: 1) daily preventive zinc supplements provided as dispersible zinc tablets plus oral rehydration salts (ORS) and placebo tablets for diarrhea (Group Prev-ZnTab); 2) daily preventive zinc supplements provided as MNP plus ORS and placebo tablets for diarrhea (Group Prev-ZnMNP); 3) daily placebo preventive supplements provided as dispersible tablets plus ORS and dispersible zinc tablets for diarrhea (Group Ther-ZnTab); and 4) daily placebo preventive supplements provided as powder plus ORS and placebo tablets for diarrhea (Control Group) (Table 1).

**Table 1:** Type of supplementation (preventive and therapeutic) by study group^a^

Type of supplement	Study Group			
	Prev-ZnTab	Prev-ZnMNP	Ther-ZnTab	Control
Preventive supplement	Zn-containing tablet	Zn-containing MNP	Placebo tablet	Placebo powder
Therapeutic supplement for diarrhea	ORS + Placebo tablet	ORS + Placebo tablet	ORS + Zn-containing tablet	ORS + Placebo tablet

^a^*ORS* oral rehydration salts, *Prev-ZnMNP* preventive zinc multiple micronutrient powder, *Prev-ZnTab* preventive zinc dispersible tablet, *Ther-ZnTab* therapeutic zinc dispersible tablet

#### Supplement products

The preventive MNP supplement contains 15 micronutrients, using the formulation currently recommended by UNICEF, [13] except for an increase in elemental zinc from 4.1–5 mg to 10 mg per daily dose and a decrease in elemental iron from 10 mg to 6 mg per daily dose (Table 2). The rationale for the higher dose of zinc is supported by two evidence bases. Firstly, most previous studies have not detected any effect of 5 mg zinc provided in MNP or fortified porridges on plasma zinc concentrations [43–45], whereas some, but not all, studies have detected a positive response when 10 mg zinc is provided in MNP [17–19]. Secondly, a recent study found that the total absorbed zinc from MNP containing 5 mg elemental zinc barely approximated infant and young children’s estimated physiologic requirement for absorbed zinc and the authors suggested that higher doses may be needed in vulnerable populations [46]. The iron dose was reduced to 6 mg per day due to concerns that higher amounts of iron may cause adverse effects [47] and because another trial providing 6 mg iron in a home fortification product found beneficial impacts on anemia and iron status [48]. The preventive zinc dispersible tablet contains 7 mg of elemental zinc; in the only available dose-response trial of preventive zinc supplementation among young children, a daily dose of 6–7 mg zinc/d provided the maximal benefit on diarrheal disease reduction [49]. The therapeutic zinc dispersible tablet contains 20 mg elemental zinc, per WHO/UNICEF recommendations [27].

**Table 2:** Micronutrients provided in multiple micronutrient powder (MNP), in comparison to World Health Organization/Food and Agriculture Organization (WHO/FAO) Recommended Nutrient Intakes (RNI)^a,b^

Nutrient	Chemical Form	Unit	PrevZn-MNP (1 g)	WHO/FAO RNI (6–11 mo)	WHO/FAO RNI (12–23 mo)
Vitamin A	Retinol acetate	μg RE	400	400	400
Thiamin (B1)	Thiamin mononitrate	mg	0.5	0.3	0.5
Riboflavin (B2)	Riboflavin	mg	0.5	0.4	0.5
Niacin (B3)	Niacinamide	mg	6	4	6
Vitamin B6	Pyridoxine hydrochloride	mg	0.5	0.3	0.5
Folic acid (B9)	USP	μg DFE	150	80	150
Vitamin B12	Cyanocobalamin, USP	μg	0.9	0.7	0.9
Vitamin C	Ascorbic acid	mg	30	30	30
Vitamin D	Cholecalciferol (D3)	μg	5	5	5
Vitamin E	dl-α-tocopheryl acetate	mg TE	5	2.7	5
Copper	Copper sulfate, anhydrous	mg	0.56	---^c^	---^c^
Iodine	Potassium iodate	μg	90	90	90
Iron	Ferrous fumarate	mg	6	6.2–18.6^d^	3.9–11.6^d^
Selenium	Selenium selenite	μg	17	10	17
Zinc	Zinc gluconate	mg	10	2.5–8.4^e^	2.4–8.3^e^

^a^*DFE* dietary folate equivalent, *PrevZn-MNP* preventive zinc multiple micronutrient powder, *RE* retinol equivalent, *RNI* recommended nutrient intake, *TE* tocopheryl equivalents, ^b^WHO/FAO requirements are from World Health Organization/Food and Agriculture Organization (2004), ^c^No WHO/FAO RNI; ^d^WHO/FAO RNI are shown for diets with varying assumptions regarding bioavailability (15–5%); ^e^WHO/FAO RNI are shown for diets with varying levels of bioavailability (high – low)

Because the purpose of the present study is to compare different delivery products for zinc and not to compare a specific zinc compound, the chemical forms of zinc in the MNP and dispersible tablets are according to the usual form chosen by the respective producers. Specifically, the chemical form of zinc in the MNP and the dispersible tablets are zinc gluconate and zinc sulfate, respectively. The excipients are maltodextrin (MNP) and cellulose, starch, sweetener, vanilla flavor, silica, and magnesium stearate (dispersible tablet). Placebo powders and placebo dispersible tablets (preventive and therapeutic) contain excipients only. The preventive MNP and placebo powders were produced by DSM Fortitech Asia Pacific Sdn Bhd (Banting, Malaysia) in strips of five sachets, each containing a single daily dose. The dispersible tablets (preventive and therapeutic supplements and placebos) were produced by Nutriset SAS (Malaunay, France) in blister packs of 10 tablets. All supplements were coded at the time of manufacture with a pre-printed unique 2 digit code, corresponding to one of the four study arms (17, 30, 66, 84), as well as a “P” (preventive) or “T” (therapeutic). All intervention products are color-coded by study staff with one of four colors corresponding to the study arms (P17/T17 = red; P30/T30 = blue; P66/T66 = green; P84/T84 = purple) to facilitate recognition. Supplements are stored at the project field office in a secure, air-conditioned room (25 °C), with the temperature monitored daily.

#### Supplement administration

The supplementation regimen for the preventive supplements is daily, and the supplements are delivered weekly during the home visits by the morbidity surveillance workers (MSW). Daily preventive supplementation is scheduled to begin the day following enrollment. However, in the event that diarrhea is diagnosed during enrollment, supplementation begins on the first day of enrollment in compliance with the therapeutic intervention protocol. In both cases, baseline biological samples, including blood, hair and stool, are collected prior to the initiation of supplementation. Daily supplementation continues for 36 weeks; in cases where endline assessment is delayed, supplementation continues for up to 40 weeks. MSW deliver eight of the respective daily preventive supplements. The extra supplement is provided in case the subsequent weekly home visit is delayed, or in case of loss.

The therapeutic supplements are part of the diarrhea management along with ORS starting on the first day of diarrhea. One diarrhea treatment kit is given to each primary caregiver during enrollment, with instructions to store it in the home and use it for the treatment of a diarrhea episode in the study child. Each kit contains the following inside of a clearly labeled, sealed plastic bag: 10 tablets of the group-specific form of the dispersible therapeutic tablet, 3 sachets of low-osmolarity ORS (Oreda R.O, Thailand), a plastic spoon and an insert with instructions on how to use the contents of the diarrhea kit. The insert contains written instructions in Lao, as well as an image of a child with diarrhea, and symbols to indicate usage instructions. At each weekly home visit, the MSW verifies that the full diarrhea kit is available; if the diarrhea treatment kit in the home is used, missing or misplaced, a new diarrhea kit is delivered by the MSW. All MSWs are given color-coded back-up supplies of both preventive and diarrhea treatment kits, such that supplements can be rapidly replaced in the case of use or loss.

Educational sessions are conducted with primary caregivers at the end of the enrollment day to explain how to give intervention products. In addition, MSWs are required to review the instructions with caregivers at least every 4 weeks and during and after all diarrhea episodes. Caregivers also receive a poster depicting proper use of the preventive and therapeutic supplements. Focus groups, conducted with caregivers of young children during the pilot study, confirmed that MNP feeding instructions previously used in a neighboring province would be acceptable in the study area [50]. The focus groups also informed educational messages for community mobilization. For children in the Prev-ZnMNP and Control groups, caregivers are instructed to add the entire contents of the powder package into a single serving of semi-solid or mashed food after the food has been cooked and cooled sufficiently to be eaten (but within 30 min of preparation). Examples of suitable foods, such as mashed mango, banana and papaya, boiled pumpkin, and boiled egg are described during the education session and repeated during MSW visits [50]. For children in the Prev-ZnTab and Ther-ZnTab groups, caregivers are advised to mix the tablet with clean water or breast milk and spoon feed the dissolved tablet to the child 30 min before or after food consumption. All caregivers are further instructed to not provide more than one dose per day to the study child, and to not share the products with other household members. In addition, caregivers receive clear instructions to begin diarrhea treatment (ORS plus dispersible tablets) when their child has 3 or more liquid or semi-liquid stools in 24 h, and to continue dispersible tablet treatment for 10 days, until all therapeutic tablets are gone. On each day that the child has 3 or more liquid or semi-liquid stools, caregivers are advised to prepare fresh ORS and give it to the child intermittently throughout the day. For ethical reasons, all children with diarrhea persisting for > 14 days (i.e. persistent diarrhea) receive a 10 day course of unblinded “ZincFant” dispersible tablets (Nutriset, SAS), containing 20 mg zinc/tablet, irrespective of their study group assignment. Caregivers of children given “ZincFant” are instructed to temporarily stop providing the assigned study interventions to the child until the “ZincFant” course is completed.

#### Concomitant medications and referral

Children > 12 months of age who test positive for intestinal helminthes at baseline and/or at the end of the study (after the collection of stool samples) receive a standard dose of mebendazole (500 mg), according to national protocols. Children who test positive for α-or β-thalassemia receive genetic counseling in accordance with national guidelines. If a child is identified with diarrhea with complications (e.g. dysentery) or any other illness requiring medical follow up, the child is referred to the nearest health center. In these situations, caregivers are instructed to continue study interventions per protocol.

### Data collection

#### Visit schedule

**Fig. 2:**
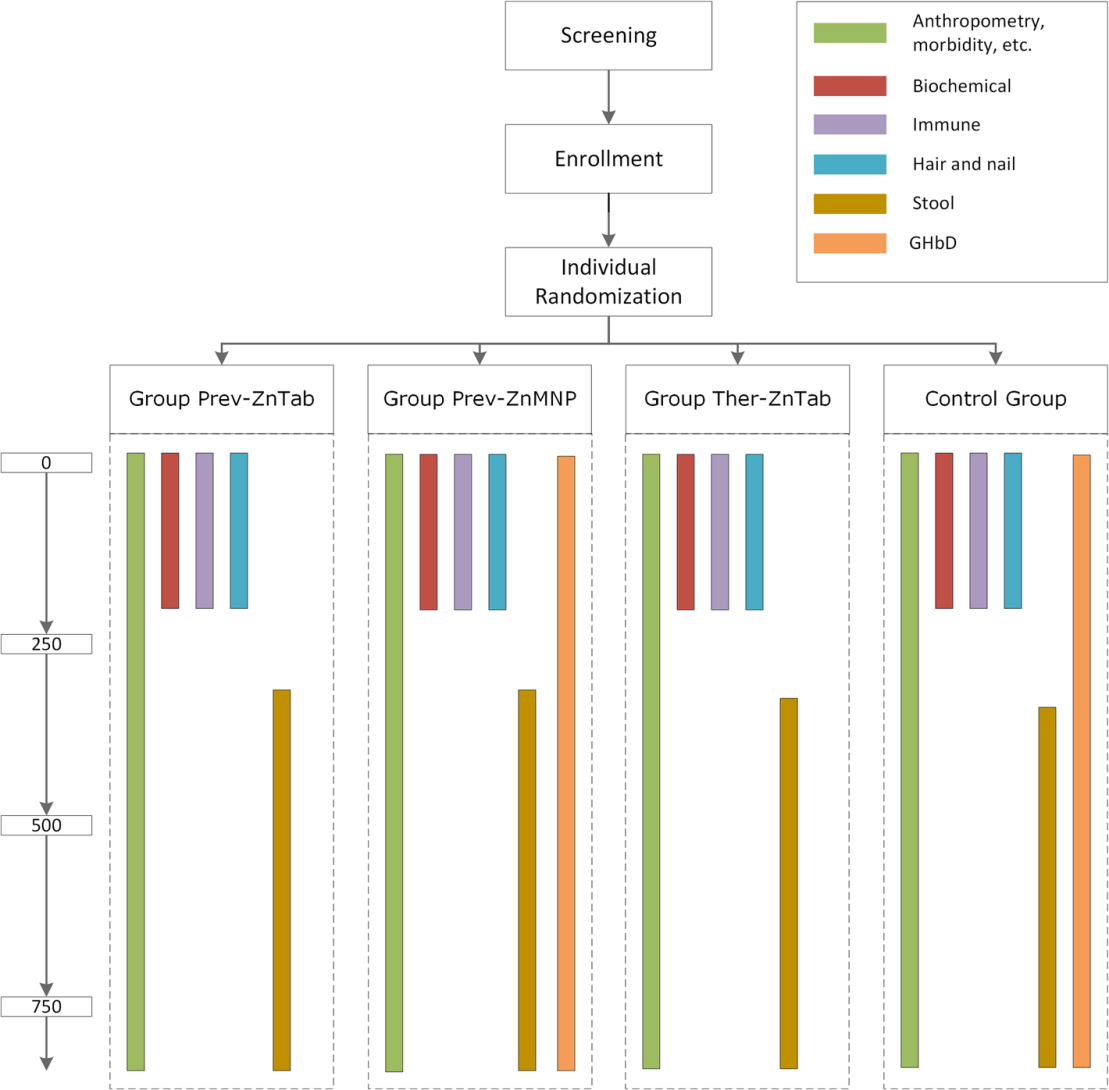
Summary of sub-samples among entire study cohort

Study measurements at different time points are listed in Table 3. In brief, children visit a centralized field site in their village for the three major data collection time points of screening/enrollment, 16–20 weeks and 32–36 weeks (baseline, mid-point and final). At the baseline and final assessments, a mobile field team measures anthropometric indicators in all children, and collects blood, stool, hair, and nail samples from sub-samples of the enrolled children (Fig. 2); an additional anthropometric measurement and hair collection is conducted at mid-point. Final biochemical assessments are conducted prior to 36 weeks to ensure that children are still consuming the supplement at time of the assessment, which is important for the measurement of plasma zinc concentrations [51], and to allow for flexibility in rescheduling the assessment if any children are ill at the time of the final assessment. All enrolled children receive in-home visits conducted weekly for 36 weeks by MSW to assess morbidity and adherence to supplementation. Additional information, collected via structured oral interviews, is collected at pre-determined intervals (Table 3).

**Table 3:**
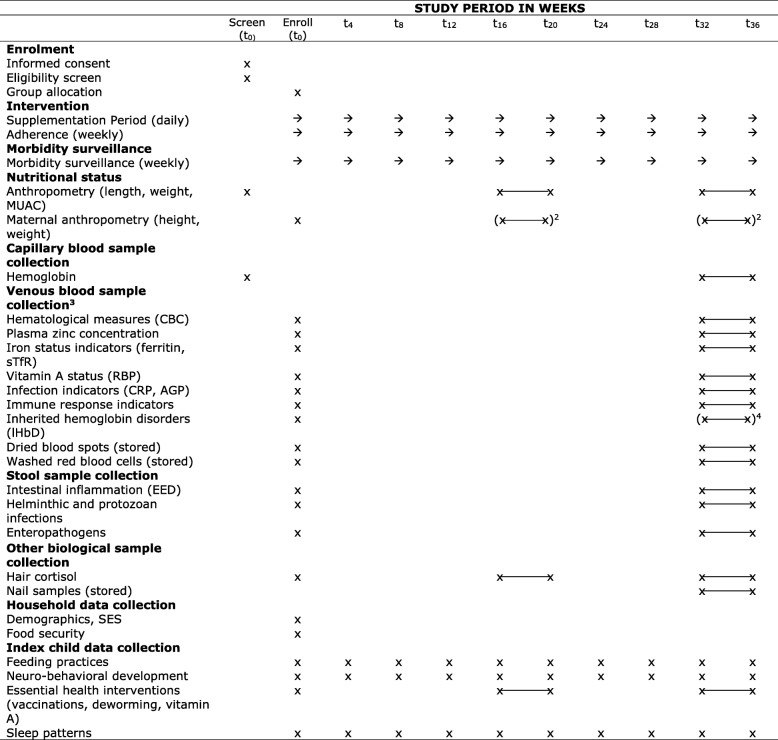
Schedule of enrollment, intervention and assessments^1^

^1^AGP, α-1-acid glycoprotein; CBC, complete blood count; CRP, c-reactive protein; EED, environmental enteric dysfunction; IHbD, inherited hemoglobin disorders; MUAC, mid-upper arm circumference; RBP, retinol binding protein; SES, socio-economic status; sTfR, soluble transferrin receptor; ^2^Maternal anthropometry assessment was attempted at 16–20 or 32–36 weeks only if not completed at baseline (i.e. alternative caregiver brought child to assessment site); ^3^Venous blood sample collection in biochemical, immune and IHbD sub-samples. Plasma zinc, iron status, vitamin A status and inflammation assessed in biochemical sub-sample. CBC and immune response indicators assessed in immune sub-sample. CBC, iron status, vitamin A status, inflammation, and IHbD assessed in IHbD sub-sample; ^4^IHbD assessment was attempted at 32–36 only if not completed at baseline

#### Morbidity

At each weekly home visit, systematic symptom-based recalls are used by the MSW to obtain information on the presence and severity of selected symptoms of pre-defined diseases for each day of the preceding week. Caregivers are given a weekly pictorial chart, which allows them to record the number of liquid or semi-liquid stools and the presence of other selected symptoms of illness, and are used to facilitate recall. Specifically, the MSW collects information about stool number and consistency, the presence of fever, vomiting, cough, respiratory difficulties, nasal discharge, skin rash, depressed appetite, and any other symptoms of concern to the caregiver. In addition, any visits to a medical professional, hospitalization, and concomitant medications are recorded. Axillary temperature is measured whenever fever is reported within the previous 24 h of the time of the home visit, and once every 4 weeks, regardless of reported fever status. The MSW provides referrals to government health clinics as necessary. If no one is home at the time of the scheduled weekly home visit, the MSW revisits the household, either later the same day or on the next day that a caregiver is expected to be available, in an attempt to complete the interview before the next scheduled home visit. In the case of longer absences, missed interviews are documented in MSW reports and weekly visits are resumed as soon as possible. Exit interviews are completed either on the final scheduled home-visit, or whenever a child is determined to be a loss to follow-up.

When a caregiver reports that a child has had > 3 liquid or semi-liquid stools within a 24 h period in the interval since the last home visit, the MSW reminds the caregiver about the diarrhea management protocol and opens a diarrhea case report to monitor symptoms and adherence to treatment. The case report is used to track the duration of diarrhea episodes, and remains open until the 10 day treatment regimen is completed, provided the diarrhea has resolved. If the diarrhea persists for >10 days, children continue to receive therapeutic supplements based on their study group, and the case report is closed upon the resolution of diarrhea. Case reports are used to closely monitor children at risk of persistent diarrhea, and provide unblinded “ZincFant” containing 20 mg zinc/day as necessary.

#### Adherence

Adherence to the interventions (preventive and therapeutic supplements) is assessed indirectly, using participant reporting and product disappearance rate. Caregivers are able to record consumption of the preventive and therapeutic supplements on the aforementioned weekly pictorial charts. During the weekly morbidity surveillance visits, MSWs interview caregivers regarding supplement consumption, using the pictorial chart to facilitate recall. In addition, the MSW collects, counts and records the number of both empty and unused preventive and therapeutic supplements (sachets and/or blister packs), during each weekly visit.

#### Anthropometry

Child anthropometric data are collected by trained, standardized anthropometrists at baseline, mid-point and endline assessments. In addition, maternal anthropometric data are collected at baseline; if the mother is unavailable at baseline, attempts are made to collect her anthropometric data at the mid-point or endline assessment. Protocols established by the Food and Nutrition Technical Assistance Project are followed for all measurements [52]. Unclothed children, and lightly clothed mothers, are weighed to 20 g and 50 g precision, respectively (SECA 383 and SECA 874). If children are too agitated, they are weighed in the caregiver’s arms on a digital floor scale with a mother-child weighing function (SECA 874), to 50 g precision. Children’s recumbent length (SECA 416), mid-upper arm circumference (MUAC, left arm; ShorrTape© Measuring Tape, Weigh and Measure, Olney, MD), and maternal height (SECA 213) are measured to 0.1 cm precision. All measurements are collected in duplicate and the average of the two measurements is calculated per child for each outcome. If measurements differ by greater than 0.1 kg (weight), or 0.5 cm (length, height, MUAC), the measurement is repeated a third time and the two closest measurements within 0.1 kg or 0.5 cm of one another are retained. If no two measurements meet this criteria, duplicate measurements are repeated. Two teams of two anthropometrists are systematically rotated to different communities for baseline, midpoint and endline assessments to minimize potential measurement bias, and are systematically re-standardized approximately every 2 months [53].

#### Biological sample collection and laboratory analyses

##### *Capillary and venous blood collection*

**Fig. 3:**
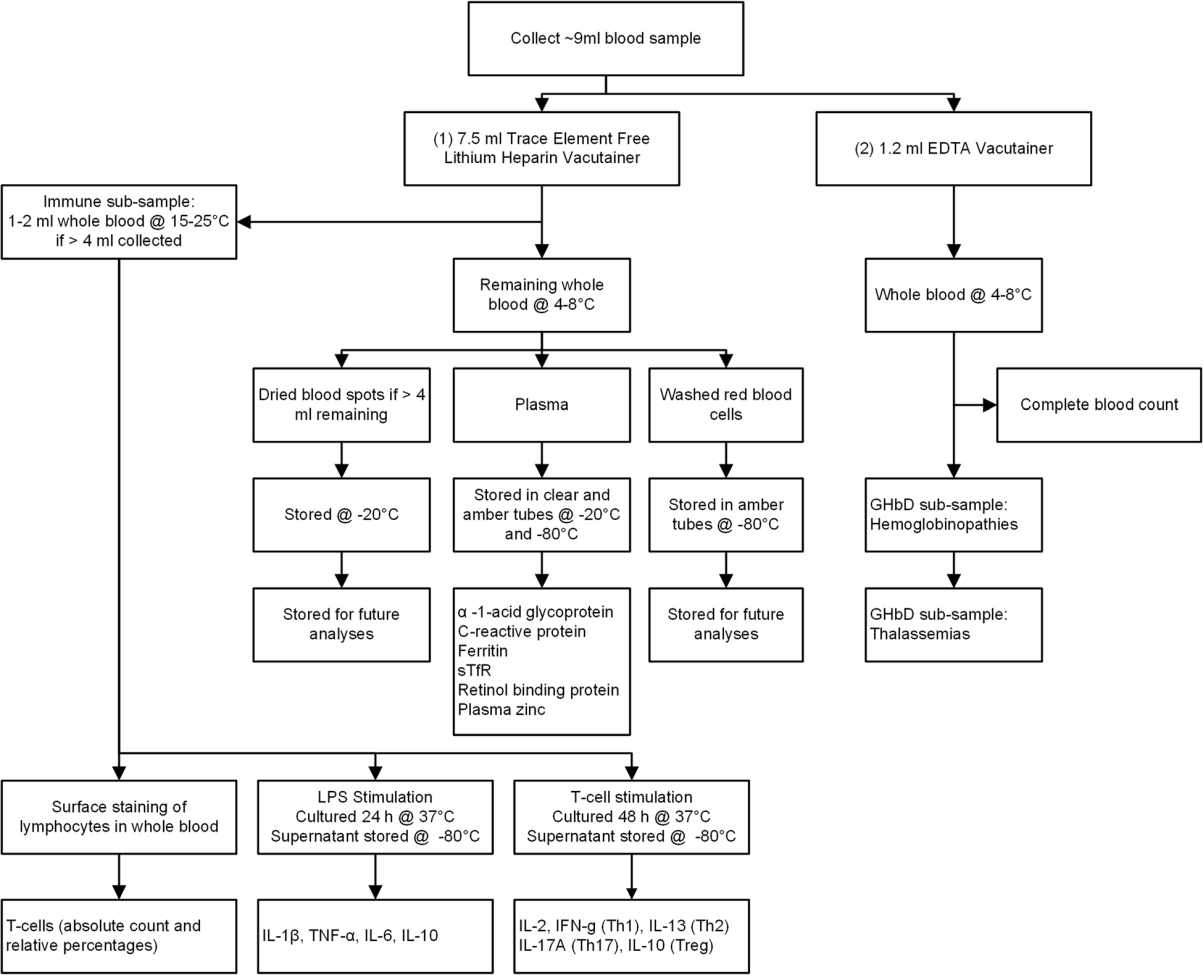
Flow chart of blood collection and analysis

Hemoglobin concentrations are measured at baseline and endline from capillary blood samples using HemoCue® Hb 301 (HemoCue AB, Angelholm, Sweden). Venous blood samples are collected from children in the biochemical, immune and IHbD sub-samples, by trained nurses and laboratory technicians at baseline and endline assessments (Fig. 3). When a child is acutely ill with diarrhea within 24 h of the scheduled blood drawing, or presents with fever (measured axillary temperature > 37.5 °C), baseline venous blood collection does not occur; venous blood collection at endline is postponed until after symptoms have resolved. For each blood-sampling event, ≤ 9 ml of blood is drawn from an antecubital, dorsal metacarpal, or great saphenous vein according to procedures recommended by IZiNCG [54]. Samples to be analyzed for plasma zinc are typically collected in the morning before 12 pm and the time of blood draw is recorded. The blood is collected firstly into an evacuated, trace element-free 7.5 ml polyethylene blood collection tube containing lithium heparin (LiHep) and secondly into an evacuated 1.2 ml polyethylene blood collection tube containing EDTA (Sarstedt AG & Co, Numbrecht, Germany; ref. 01.1604.400 and 06.1666.100, respectively). If > 4 ml is collected in the LiHep tubes, and the participant is in the biochemical sub-sample, 1–2 ml of the collected whole blood sample is immediately aliquoted into a 2 ml microcentrifuge tube, and stored at 15–25 °C. These samples are transported daily to Nakhon Phanom Hospital (NPH; Nakhon Phanom, Thailand) for assessment of immune function (immune sub-sample). The remaining heparinized blood is stored at 4–8 °C until transport to the field laboratory. The EDTA blood samples are stored at 4–8 °C, and are either transported daily (immune sub-sample) or weekly (remaining IHbD sub-sample) to NPH.

If > 4 ml whole blood remains in the LiHep tubes, 50 μl of blood is applied to each circle (× 5) of a dried blood spot (DBS) card (Whatman 903 Protein Saver). DBS cards are dried for 24 h in the field laboratory, and then stored at − 20 °C in small batches in Ziploc bags containing desiccants. The remaining heparinized blood is centrifuged within 8 h of collection at 1097 x g (3100 RPM) for 10 min (PowerSpin Centrifuge Model LX C856; United Products & Instruments, Inc., Dayton, NJ). Plasma is aliquoted into 0.2–1.5 ml pre-labeled microcentrifuge tubes (clear and amber, depending on planned analyses), and stored at − 20 °C or − 80 °C. Packed erythrocytes are washed three times in ~ 5 ml physiological saline (9 g/L NaCl), with care taken to remove the buffy coat. To hemolyze the cells, 0.5 ml distilled water is added to 0.5 ml aliquots of red blood cells in pre-labeled 1.5 ml amber microcentrifuge tubes, and vortexed for 1 min. Samples are stored at − 20 °C for up to 1 week, and then stored at − 80 °C for the duration of the field study. All samples stored at − 20 to − 80 °C are shipped on dry ice to their respective laboratories for analysis.

Complete blood counts (CBC) are performed on venous blood samples collected in EDTA tubes and stored at 4–8 °C for < 24 h prior to analysis by using one of two automated hematology analyzers [(XT-1800, Sysmex; Sysmex Corporation) at NPH in Nakhon Phanom, Thailand and (BC-3000Plus, Mindray Medical International Ltd.) at the project field laboratory in Nongbok, Lao PDR]. To measure cytokine production following stimulation of the innate and adaptive immune systems, two sets of heparinized blood cultures (maintained at 37 °C in 5% CO_2_; with appropriate negative control cultures) are initiated at NPH. Innate stimulation involves bacterial lipopolysaccharide (LPS) and measurement of cytokines including IL-1β, TNF-α, IL-6 and IL-10 in 24 h culture supernatants. Adaptive stimulation uses antibodies targeting T-cells (anti-CD3 plus anti-CD28) and measurement of cytokines including IL-2, IFN-γ (Th1), IL-13 (Th2), IL-17A (Th17) and IL-10 (Treg) in 48 h supernatants. In addition, whole blood is processed at NPH to determine absolute counts and percentages of T-cell subsets (including total and memory CD4 and CD8 T-cells, and Treg cells) by flow cytometric analysis (FACSCalibur) performed at Khon Kaen University (Khon Kaen, Thailand). Plasma zinc concentrations will be analyzed by inductively couple plasma optical emission spectrophotometry (ICP-OES Agilent 5100 SVDV, Santa Clara, CA) at the Children’s Hospital of Oakland Research Institute (CHORI; Oakland, CA), as described previously [55]. Plasma ferritin, soluble transferrin receptor (sTfR), retinol binding protein (RBP), C-reactive protein (CRP) and α-1-acid glycoprotein (AGP) concentrations will be measured in duplicate using a combined sandwich enzyme-linked immunosorbent assay (ELISA) technique at VitMin Lab (Willstaett, Germany) [56].

IHbD will be detected using hemoglobin electrophoresis at NPH and polymerase chain reaction (PCR) techniques at Khon Kaen University (Thailand) [57]. The types and levels of the following hemoglobin fractions will be determined: normal hemoglobin (A, A_2_, F), hemoglobin variants [E, H, Constant Spring (CS)], and α- and β-thalassemia.

##### Stool collection and assessment of intestinal parasite infection and environmental enteric dysfunction

Collection of stool samples is attempted on two consecutive days from children in the stool sub-sample at both baseline and endline; samples are collected in duplicate at each time point to minimize failure to detect low-intensity helminth infection. Disposable diapers are distributed to caregivers with the instructions to place the first diaper on the child immediately before sleep on the evenings prior to the days of stool collection. Caregivers are instructed to return to the study site the following day with all diapers containing stool, and to not bathe the child the morning of collection. At baseline, the 2 day collection requirement necessitates that caregivers be given diapers during the community sensitization session, 1 day prior to the baseline assessment. Stool samples are only stored and analyzed from children whose parents give oral consent at the community sensitization session, written informed consent on the day of enrollment and are eligible to participate in the study. Children presenting with diarrhea at enrollment have less than 2 days of stool collection at baseline, because preventive and therapeutic supplementation are begun immediately following diagnosis.

For immediate stool sample processing a mobile laboratory is set up. Trained laboratory technicians aliquot stool from the most recent defecation into two stool containers; the approximate time of defecation, based on caregiver report, is recorded. From the first stool container, samples are immediately prepared for intestinal parasite analysis by Kato-Katz (KK) technique and formalin-ethyl acetate concentration technique (FECT). In brief, duplicate KK thick smears are prepared according to standard procedures using sieved stool samples [58, 59] and allowed to clear for 15 min prior to examination under light microscopy at the mobile assessment site. Helminth eggs are counted and recorded for each species separately. In addition, the second stool sample is fixed using sodium acetate acetic acid formalin (SAF) solution and will be processed according to established FECT protocols and analyzed at NIOPH in Vientiane, Lao PDR [60]. From the second stool container, laboratory technicians aliquot fecal samples into 1.5 ml amber microcentrifuge tubes and 2.0 ml clear PCR performance tested microcentrifuge tubes and immediately store at − 18 °C in a portable freezer (CF-025, Dometic, Solna, Sweden). Samples are subsequently stored at − 20 °C for up to 7 days, and at − 20 °C or − 80 °C until analysis. Stool samples will be shipped on dry ice to the University of California, Davis, where they will be analyzed for indicators of environmental enteric dysfunction (EED). In particular, fecal calprotectin and neopterin concentrations will be measured using commercially available ELISA kits, following the manufacturer’s instructions.

In addition, the scotch test, for the detection of pinworm, is performed on either day 1 or day 2 of stool collection among those children whose caregivers followed the instructions to not wash the child prior to arrival at the mobile assessment site. The adhesive side of clear cellophane tape (~ 3–5 cm) is applied against the skin across the child’s anal opening with firm, even pressure and then transferred to a microscope slide. Slides are observed under a microscope for the presence of pinworm eggs by a trained laboratory technician at the mobile assessment site.

##### Hair and nail collection

Approximately 10–20 mg of hair is cut as close to the scalp as possible from the center of the nape of the neck (~ 2 cm above hair line), or a nearby location, in case of insufficient hair. In case of longer hair (> 2 cm), the hair is taped to an index card using cellophane tape and the side closest to the scalp is identified. All hair is stored in small paper envelopes within Ziploc bags, at room temperature. Finger and toenails are cut using infant safety nail clippers and stored in small paper envelopes within Ziploc bags at ambient temperature in an airtight container, with desiccants. Hair cortisol concentrations will be quantified using an ELISA designed to quantify free unbound cortisol (Salimetrics, State College, PA, USA) at the University of Colorado, Boulder.

##### Specimen banking

Plasma, red blood cells, DBS, stool, hair and nail samples will be stored in the above described conditions at the University of California, Davis for future analyses of indicators of interest, including novel markers of zinc and other markers of nutritional status, growth and infection.

#### Questionnaires

Trained personnel administer all questionnaires as structured oral interviews with primary caregivers, using electronic tablets for data collection. At baseline, data on demographic and socio-economic status (education, occupation, ethnicity, household size and composition, housing quality, hygiene and sanitation, household assets, household land ownership) and food security [Household Food Insecurity Access Scale (HFIAS)] [61] are collected. Neurobehavioral development is assessed every 4 weeks by maternal report and by direct observation by the MSW of eleven indicators of gross motor, personal-social and language developmental milestones. Six of the gross motor milestones (sitting without support, hands-and-knees crawling, standing with assistance, walking with assistance, standing alone, walking alone) were evaluated in the WHO Motor Development Study, and have established windows of achievement [62]; the remaining developmental milestones evaluated in this study were selected from the Denver Development Screening Test 2 (running, pronouncing single words, waving goodbye, eating by self, and drinking from a cup) [63]. Information on infant and young child feeding practices (breastfeeding, formula feeding, 24 h and 7d food frequency questionnaire) is collected every 4 weeks, and used to calculate selected WHO infant and young child feeding (IYCF) indicators [64, 65]. The dietary assessment questionnaire also includes additional questions to ascertain sanitation indicators, and reported receipt of high-dose vitamin A supplements and deworming medication. The Brief Infant-Child Sleep Questionnaire form is administered monthly to assess the impact of study interventions on sleep patterns in children [66]. History of essential health interventions including vaccines, vitamin A supplementation and deworming, as indicated on child immunization cards and health center WHO Expanded Programme on Immunization (EPI) registries is also recorded at baseline, mid-point, and final assessments.

### Data management and statistics

#### Electronic data collection and management

All data are collected electronically using a CommCare-HQ application (Dimagi, Boston, MA), a secured, open source, and cloud-based mobile platform. All data collection forms were developed in a customized application by study personnel, and include pre-determined logic checks and range validations. Forms are deployed onto Samsung tablets (Samsung Galaxy Tab. 3 V) equipped for mobile internet access (via providers Lao Telecom and Unitel); all data collected are synchronized onto a central server. Study staff are extensively trained on the use of tablets and the CommCare application and data integrity. Geo-coded data are collected to verify the location of home-based interviews. A data monitoring protocol is implemented weekly to evaluate the proportion of expected data synced to the server (per-child and per-MSW). A data-driven supervision approach is used to enable targeted supervision of MSWs, supervisors, villages or study children. Children with either delayed data submission, or a low adherence rate, are identified and flagged on a weekly basis for scheduled home visits by a senior supervisor. The supervisor and MSWs of all flagged children are also visited to investigate and resolve the issues identified. MSW supervisors are required to observe interviews for at least three randomly selected children per MSW per week. In addition supervisors conduct repeated interviews for one randomly selected child per MSW per week.

#### Trial power and sample size

Effect sizes of different outcomes were determined based on available literature and considerations of public health importance, as described below (Table 4). Primary outcomes of physical growth (length and weight) and incidence of diarrhea had the smallest estimated effect sizes, and thus were the primary determinants of the overall trial sample size.

**Table 4:** Sample size calculations to assess intervention impact on primary and secondary outcomes

Variable	Detectable difference	β^a^	Unadjusted sample size per treatment group	Attrition	Adjusted sample size per treatment group^b^	Total sample size, four treatment groups
Primary outcomes						
Length and length-for-age-Z-score	0.20 SD	0.10	710	15%	850	3400
Weight and weight-for-age-Z-score	0.20 SD	0.10	710	15%	850	3400
Incidence of diarrhea	0.20 SD	0.10	710	15%	850	3400
Biochemical indicators of micronutrient status^c^	0.40 SD	0.20	133	30%	190	760
Indicators of innate and adaptive immune response	0.50 SD	0.20	88	30%	125	500
Secondary outcomes						
Intestinal parasite burden	0.30 SD	0.20	244	20%	305	1220
Indicators of environmental enteric dysfunction	0.35 SD	0.20	179	20%	225	900
Hair cortisol	0.40 SD	0.20	133	30%	190	760

^a^Power used for sample size calculation with a significance of α = 0.05

^b^Adjusted for attrition and rounded up

^c^Hemoglobin concentration was used as a screening criteria for study enrollment; thus the hemoglobin concentration of all children enrolled in the study was assessed and sample size calculations were not completed for this primary outcome

An effect size of 0.20 SD (*α* = 0.05, *β* = 0.10) for group-wise comparisons of diarrhea incidence and physical growth (length and weight) in the four-arm trial was considered to be of public health importance, and is consistent with what has been observed for diarrhea incidence and growth outcomes in previous preventive zinc supplementation trials [1–3, 8, 67]. To allow the detection of potential adverse effects of the intervention on diarrhea incidence and physical growth, a power of 90% (i.e. *β* = 0.10) was used for these outcomes. A total of 710 children are needed in each of the four treatment groups. Allowing for 15% attrition, we will need to enroll 835 children per group, which we have rounded up to 850 per group, or a total of 3400 children.

The sample size estimate for biochemical outcomes (plasma zinc, ferritin, sTfR, RBP, CRP, and AGP concentrations) and hair cortisol concentrations was based on the number of children needed in each group (*n* = 133) to detect treatment-related differences having an effect size of 0.4 SD (*α* = 0.05, *β* = 0.20) [2]. This target sample size was increased to a total of 760 children in the four intervention groups to allow for 30% attrition. Hemoglobin concentration is used as a screening criteria for study enrollment, and thus will be assessed for all children. Indicators of immune response will be determined from as many children in the biochemical outcomes sub-sample as possible, accounting for venous blood draw failures and scheduling constraints to transport the samples to NPH in time for processing; the target sample size is *n* = 125 children per study group. Although the literature on the effect of zinc supplementation on immune function is limited, a comparable study observed statistically significant treatment related differences in the naïve:memory T cell ratio with 50–75 children per intervention group [68].

All children in the Prev-ZnMNP and Control study groups (i.e. children who receive supplemental iron and children in the supplemented control group; *n* = 1700) will be screened for IHbD; in addition, biochemical outcomes (plasma ferritin, sTfR, RBP, CRP and AGP) will be determined at baseline and endline in all Prev-ZnMNP and Control children. This planned sample size is based on the full sample size available because the primary purpose of this analysis is to examine potential modifying effects of IHbD and iron status on the response to the intervention. The sample size of each of these two groups in the main trial (*n* = 850) will allow us to detect a difference in effect size of ~ 0.35 for the modifying effect of iron status or IHbD on the main effects (*α* = 0.05, *β* = 0.10, attrition = 15%).

The sample size estimates for the stool sub-sample were based on the number of children needed in each group (parasite burden, *n* = 244 and indicators of EED, *n* = 179) to detect treatment-related differences having effect sizes of 0.30 and 0.35, respectively (*α* = 0.05, *β* = 0.20) [22, 69]. The target sample sizes were increased to a total of 1220 (parasite burden) and 900 (indicators of EED) in the four intervention groups to allow for 20% attrition. However, due to the difficulties in obtaining duplicate stool samples from young children, which were encountered in the first few weeks of stool collection, stool sample collection will be attempted in a convenience sample of 2000 children, in order to meet the aforementioned target sample sizes.

Due to the large study area causing logistical challenges (e.g. daily transport of samples for immune response indicators from the field sites to NPH across the Mekong River via ferry), participants are selected for separate subgroup analyses based on logistical feasibility. In particular, biochemical and immune response outcomes are determined only in children enrolled in the study from the health districts closest to the study office (Xebangfai and Nongbok) until the sample size is met (biochemical and immune sub-samples). In addition, due to the timing and allocation of additional research funding, stool samples are collected from all children enrolled later in the study from the Mahaxay, Xaibouathong, and Yommalath districts, and a second round of enrollment from Xebangfai and Nongbok (stool sub-sample) (Fig. 2).

#### Outcomes

Primary study outcomes are physical growth (length and weight), incidence of diarrhea, hemoglobin and micronutrient status and innate and adaptive immune function. Secondary outcomes include mid-upper arm circumference, acquisition of developmental milestones, hair cortisol concentration, indicators of EED and intestinal parasite burden. Evidence of adverse effects on morbidity and growth, and severe adverse events (death and hospitalization), will be monitored as secondary outcomes for safety.

#### Definitions of variables

For daily preventive supplements, caregiver reported adherence will be calculated using two approaches: 1) as the number of days the preventive supplement was reportedly consumed divided by the number of child observation days, and 2) as the number of days the supplement was reportedly consumed divided by the number of days the child had access to the supplement. Daily disappearance rate will be calculated as the difference between the number of distributed preventive supplements and the unused packages divided by the number of observation days. For therapeutic supplements, caregiver reported adherence will be calculated based on the number of child–at risk–days (10 days per episode of diarrhea).

Diarrhea will be defined as the passage of ≥ 3 loose or liquid stools per day (24 h). Severe diarrhea will be defined as ≥ 6 liquid or semi-liquid stools in a 24 h period. Persistent diarrhea will be defined as diarrhea lasting >14 days. Dysentery will be defined as diarrhea plus blood in the stool. An episode of diarrhea will defined as the period that starts on the day the child first has diarrhea following a diarrhea-free period of at least 2 days, and ends on the last day the child has diarrhea that was followed by at least 2 days without diarrhea.

Incidence of diarrhea will be defined as the number of new episodes of diarrhea divided by the total number of child-at risk-days. Duration of acute diarrhea will be calculated as the number of days per episode. Longitudinal prevalence of diarrhea will be defined as the number of days with diarrhea divided by the total number of child-at risk-days.

Fever will be defined as: 1) any fever reported by the caregiver, whether or not the fever was confirmed by measured temperature during the last 24 h; or 2) any elevated measured axillary temperature (> 37.5 °C). An episode of fever will be defined as for diarrhea. Acute lower respiratory illness (ALRI) will be defined as any episode in which the caregiver reported cough with respiratory difficulties (wheezing/stridor or chest in-drawing). An episode of ALRI ends on the last day the child has ALRI that is followed by at least 3 days free of respiratory distress. Acute upper respiratory illness (AURI) will be defined as any episode in which the caregiver reported cough and a purulent nasal discharge. An episode ends on the last day the child has AURI that is followed by at least 7 days free of purulent nasal discharge.

Weight-for-age (WAZ), length-for-age (LAZ), weight-for-length (WLZ) and mid-upper-arm-circumference-for-age (MUACZ) Z scores will be calculated according to the WHO growth standards [70, 71]. Stunting, underweight and wasting will be defined as <− 2 SD LAZ, WAZ and WLZ and MUACZ, respectively.

Micronutrient deficiencies and anemia will be defined based on the following cut-offs: (a) zinc deficiency: plasma zinc < 65 μg/dL, (b) anemia: Hb < 110 g/L, (c) iron deficiency: ferritin < 12 μg/L or sTfR ≥ 8.3 mg/L, (d) iron deficiency anemia: Hb < 110 g/L and either ferritin < 12 μg/L or sTfR ≥ 8.3 mg/L. A study-specific cut-off value for RBP, corresponding to a retinol concentration of 0.70 μmol/L and indicative of vitamin A deficiency will be determined [48]. A linear regression correction (RC) approach will be used to adjust plasma zinc, ferritin and RBP, and if appropriate sTfR, concentrations for elevated CRP and AGP concentrations to remove the confounding effect of sub-clinical infection and inflammation on the assessment of nutritional status and deficiency [72, 73].

### Interim data monitoring and analysis

Adverse events are monitored through the weekly child health assessments conducted by the MSWs. All suspected adverse events are documented and reviewed by a study physician, and reported according to standard operating procedures of the IRBs at the Lao PDR NIOPH and UC Davis. Serious adverse events (SAE; e.g. events resulting in inpatient hospitalization, persistent or significant injury, or death) are reported at regular intervals to the UC Davis and NIOPH PIs. The UC Davis PI reports *probably* or *definitely* related (> 50%) SAE to the UC Davis IRB within five business days. SAE determined to be *not likely* related (< 50%) are reported to the UC Davis IRB at the annual review.

A DSMB of four independent experts was established to monitor enrollment, compliance with study protocol, attrition, patient safety (serious adverse events) and treatment efficacy data (morbidity incidence) while the trial is ongoing. The DSMB reviews a detailed report and meets four times over the course of the study, with the ability to call for an additional review at any point during the conduct of the study. Interim analyses of SAE are done only for safety considerations; there are no formal interim analyses of efficacy planned for this trial. All information is presented to the DSMB stratified by blinded treatment group assignment; the DSMB has the authority to request an unblinding of the treatment groups and suggest stopping the trial if deemed appropriate.

#### Analysis plan

Statistical analysis plans will be created prior to analysis of each outcome or group of outcomes and will be available online for review [74]. To reduce potential bias and ensure blinding throughout the statistical analyses and interpretation of results, two strategies will be employed. First, treatment groups will remain masked until all the statistical analyses of all primary, and selected secondary outcomes, are completed and consensus is reached on the main conclusions. Separate analytical databases will be created for each research team and new masked treatment group codes will be assigned. As such, unblinding can occur once conclusions are drawn for a specific manuscript, without revealing the original four group codes.

The Lao Zinc Study is a study with many collaborators of diverse expertise and additional research questions will likely be addressed beyond the aforementioned primary and secondary outcomes. Although each research team will develop their own statistical analysis plans, we will use the following general approach. All models will include district and child age at enrolment as study control covariates to account for the known regional variation across the study site and the wide age range at enrolment. When baseline measurements exist for an outcome, they will be included in relevant models for that specific outcome, unless inclusion results in a substantial loss of sample size (e.g. > 10%) [75].

In the primary analyses, an intention-to-treat, complete case approach will be used for primary conclusions [76]. However, because the trial is designed as a mixed efficacy-effectiveness approach, with known biological relationships between the intervention product and the outcomes, per protocol comparisons will also be conducted, accounting for compliance to both preventive and therapeutic zinc supplementation regimens. Per protocol criteria will be pre-specified in each statistical analysis plan prior to analysis and unblinding.

Assessing the effect of the intervention will begin with an initial likelihood-ratio or F-test of the global null hypothesis of no difference between treatment groups. If the global null hypothesis is rejected at the 0.05 level then six post-hoc pairwise comparisons of the treatment groups will be performed while controlling for multiple testing (e.g. Tukey-Kramer method, Sidak correction). This method will be performed once in unadjusted models which contain only treatment, study control covariates, and baseline measurement (as applicable). The same method will be repeated in adjusted models with the addition of pre-specified covariates that are significantly associated with the outcome at a 0.1 significance level. Pre-specified effect modifiers will be assessed with an interaction term in the regression model. Significant interactions (*p* < 0.1) will be further examined with stratified analyses, estimation of separate regression lines, or estimation of adjusted means at key points of the covariate, in order to understand the nature of the effect modification.

Children who received 20 mg zinc supplements for persistent diarrhea will be grouped with their respective intervention group for primary analyses of treatment effects. Impacts of the additional zinc may be explored in secondary analyses but since zinc treatment is a facet of the intervention, analyzing these study participants differently is not of primary interest.

### Trial organization

The Lao Zinc Study is led by Dr. Sonja Hess (UC Davis) and Dr. Sengchanh Kounnavong (NIOPH), who serve as PIs. Technical support and input into various aspects of the study implementation, laboratory analyses, statistical analyses and result interpretation is provided by numerous experts and their research teams from different institutions. Two UC Davis post-doctoral research fellows are hired as scientific field coordinators to manage and supervise field implementation together with NIOPH researchers (core coordination team). Prior to the study start, a workshop was held between the UC Davis and NIOPH teams to review the study protocol, and reach consensus on study implementation, standard operational procedures and best practices.

The research site has a large staff of trained research personnel including five physicians, seventeen nurses, phlebotomists and lab technicians, six anthropometrists, 26 morbidity field supervisors, 98 morbidity surveillance workers, and 12 logistical and administrative staff. Most field staff are hired locally in Khammouane Province with the help of provincial and district level health authorities. Multiple targeted training sessions (1–3 weeks in duration), are held to train candidates on their respective tasks. Typically, more candidates are trained than required, and the top performers are selected for employment; this is considered particularly important for the anthropometric teams. Weekly supervisory visits are implemented by the core coordination team to ensure that all study activities are implemented following the standard operational procedures.

## Discussion

### Innovation and significance

Many countries are now rolling out large-scale programs to include therapeutic zinc supplementation in the treatment of childhood diarrhea, but few have established programs demonstrated to be effective in the prevention of zinc deficiency. It is possible that once an efficacious preventive zinc deficiency program is implemented, using either zinc supplements or MNP, the same relatively low dose of zinc in these products could be provided continuously during diarrhea episodes, yielding the same or greater benefits than would occur with the currently recommended therapeutic zinc dose of 20 mg Zn/d. On the other hand, if this study shows there is no benefit of preventive zinc supplementation or MNP for children’s overall morbidity reduction or enhanced physical growth, zinc intervention programs could be limited to provision of supplements for diarrhea treatment only. To this end, the present study is designed to compare both the zinc delivery plan (i.e., preventive versus therapeutic supplementation) as well as the form of delivering zinc (i.e., as a dispersible tablet given between meals or as a MNP given with meals) and to permit assessment of any adverse effects of MNP on the incidence of diarrhea. The Lao Zinc Study is a scientifically rigorous randomized controlled trial with a large sample size that will offer unique insights into preventive zinc and MNP supplements, and therapeutic zinc regimens in a low-income setting where there is a high prevalence of stunting and infectious morbidity (diarrhea), but minimal malaria incidence [31, 77]. It is anticipated that this study will provide evidence to the government of Lao PDR, and other similar countries, on how best to deliver supplemental zinc to prevent zinc deficiency and reduce the severity of diarrhea-related health complications.

### Risks

One possible risk of participation is an increased incidence of diarrhea among children who receive MNP, as previously found in a study in Pakistan [19]. However, a recent Cochrane review reported no significant differences in diarrhea, respiratory tract infections and malaria outcomes between those who received either iron-containing MNP or placebo. Although these results must be interpreted with caution (very few of the available studies actively assessed morbidity outcomes and the majority of those studies were not adequately powered to detect adverse events) [15], the current worldwide consensus is that the proven benefit of MNP in anemia prevention outweighs the still uncertain risk of a possibly increased incidence of morbidity, in settings where measures to prevent, diagnose and treat infectious diseases are in place. Another potential risk of the study is related to the fact that children in the control group will not receive therapeutic zinc supplementation. However, these children will receive ORS for the treatment of diarrhea, which is the current standard of care in Lao PDR, so they should not be at an increased risk of complications from the illness in this context. To mitigate the aforementioned potential risks, children will be visited weekly by MSWs, persistent diarrhea will be treated with a 10 day course of therapeutic zinc supplementation (20 mg Zn/d), and children identified with symptoms other than uncomplicated diarrhea, fever and respiratory infection will be referred to local health clinics for medical evaluation as required.

### Knowledge translations

The results will be summarized for publication in peer-reviewed scientific journals. As soon as preliminary results become available, they will be disseminated in Lao PDR locally and nationally via the local Advisory Committee and at the annual National Health Research Forum. The findings will be used to motivate discussions about zinc supplementation within the MOH and local non-governmental organizations (NGOs) that implement nutrition and diarrhea control programs. We expect that NIOPH’s leadership and the inclusion of governmental authorities, international partners and NGOs in the local Advisory Committee, will help ensure the results of the trial will be used as quickly as possible to inform into relevant national health policy and public health programs.

### Trial status

Ongoing at time of initial submission of manuscript; recruitment was completed August 2016, primary data collection was completed May 2017, final data collection was completed July 2017. Laboratory and data analyses are ongoing.

## Abbreviations

AGP: α-1-acid glycoprotein
CBC: Complete blood count
CHORI: Children’s Hospital of Oakland Research Institute
CMDL: Center for Research and Development of Medical Diagnostic Laboratories
CRP: C-reactive protein
CS: Constant Spring
CZE: Capillary zone electrophoresis
DBS: Dried blood spot
EED: Environmental enteric dysfunction
EPI: Expanded Programme on Immunization
FECT: Formalin-ethyl acetate concentration technique
Hb: Hemoglobin
HFIAS: Household Food Insecurity Access Scale
HIV: Human immuno-deficiency virus
ICP-OES: Inductively couple plasma optical emission spectrophotometry
IHbD: Inherited hemoglobin disorders
IRB: Institutional review board
IYCF: Infant and young children feeding
KK: Kato-Katz
LAZ: Length-for-age Z score
LiHep: Lithium heparin
LPS: Lipopolysaccharide
MNP: Micronutrient powder
MOH: Ministry of Health
MSW: Morbidity surveillance worker
MUAC: Mid-upper arm circumference
MUACZ: Mid-upper arm circumference-for age Z score
NECHR: National Ethics Committee for Health Research
NGO: Non-governmental organization
NIOPH: National Institute of Public Health
NPH: Nakhon Phanom Hospital
ORS: Oral rehydration salts
PCR: Polymerase chain reaction
PDR: People’s Democratic Republic
PI: Principal investigator
RBP: Retinol binding protein
RPMI: Roswell Park Memorial Institute
SAE: Serious adverse event
SAF: Sodium acetate acetic acid formalin
sTfR: Soluble transferrin receptor
Th1, Th2 and Th17: T-helper type 1, 2 and 17 cells, respectively
Tregs: T-regulatory cells
WAZ: Weight-for-age Z score
WHNRC: Western Human Nutrition Research Center
WHO: World Health Organization
WLZ: Weight-for-length Z score
Zn: Zinc
